# Student, Staff, and Faculty Perspectives on Intimate Partner and Sexual Violence on 3 Public University Campuses: Protocol for the UC Speaks Up Study and Preliminary Results

**DOI:** 10.2196/31189

**Published:** 2022-04-05

**Authors:** Jennifer A Wagman, Claire Amabile, Stephanie Sumstine, Eunhee Park, Sabrina Boyce, Jay Silverman, Rebecca Fielding-Miller, Laury Oaks, Dallas Swendeman

**Affiliations:** 1 Department of Community Health Sciences Jonathan and Karin Fielding School of Public Health University of California Los Angeles Los Angeles, CA United States; 2 Department of Psychiatry & Biobehavioral Sciences Semel Institute University of California Los Angeles Los Angeles, CA United States; 3 Center on Gender Equity and Health School of Medicine University of California San Diego La Jolla, CA United States; 4 The Herbert Wertheim School of Public Health and Human Longevity Science University of California San Diego La Jolla, CA United States; 5 Department of Feminist Studies University of California Santa Barbara Santa Barbara, CA United States

**Keywords:** campus-based violence prevention, intimate partner violence, sexual violence, mixed methods research, public health approach, prevention, student-led, trauma-informed research, University of California

## Abstract

**Background:**

Intimate partner and sexual violence are pervasive public health issues on college and university campuses in the United States. Research is recommended for creating and maintaining effective, relevant, and acceptable prevention programs and response services for student survivors.

**Objective:**

The University of California (UC) Speaks Up study aims to examine factors contributing to intimate partner and sexual violence on 3 UC campuses and use the findings to develop and test interventions and policies to prevent violence, promote health, and lay the groundwork for subsequent large-scale quantitative research.

**Methods:**

A mixed methods study was conducted at UC Los Angeles, UC San Diego, and UC Santa Barbara. Phase I (2017-2020) involved a resource audit; cultural consensus modeling of students’ perceptions of sexual consent; in-depth interviews (IDIs) and focus group discussions with students to understand perceptions of campus environment related to experiences as well as prevention of and responses to violence; and IDIs with faculty, staff, and community stakeholders to investigate institutional and community arrangements influencing students’ lives and experiences. Phase II (2020-ongoing) involves IDIs with student survivors to assess the use and perceptions of campus and community services. Qualitative content analysis is used to generate substantive codes and subthemes that emerge, using a thematic analysis approach.

**Results:**

In January 2019, we conducted 149 free-listing interviews and 214 web-based surveys with undergraduate and graduate and professional students for the cultural consensus modeling. Between February 2019 and June 2019, 179 IDIs were conducted with 86 (48%) undergraduate students, 21 (11.7%) graduate and professional students, 34 (19%) staff members, 27 (15.1%) faculty members, and 11 (6.1%) community stakeholders, and 35 focus group discussions (27/35, 77% with undergraduate students and 8/35, 23% with graduate and professional students) were conducted with 201 participants. Since September 2020, 50% (15/30) of the planned student survivor interviews have been conducted. This segment of data collection was disrupted by the COVID-19 pandemic. Recruitment is ongoing.

**Conclusions:**

Data analysis and phase II data collection are ongoing. The findings will be used to develop and test interventions for preventing violence, promoting health and well-being, and ensuring that survivor services are relevant and acceptable to and meet the needs of all individuals in the campus community, including those who are typically understudied. The findings will also be used to prepare for rigorous, UC–system-wide public health prevention research.

**International Registered Report Identifier (IRRID):**

DERR1-10.2196/31189

## Introduction

### The Public Health Problem of Intimate Partner and Sexual Violence on College Campuses

Intimate partner violence (IPV) and sexual violence are pervasive public health issues on college and university campuses in the United States [1,2]. IPV is defined by the US Centers for Disease Control and Prevention as “physical violence, sexual violence, stalking, and psychological aggression (including coercive tactics) by a current or former intimate partner (i.e., spouse, boyfriend or girlfriend, dating partner, or ongoing sexual partner)” [3]. It is estimated that one-third of all college students in the United States have experienced some form of IPV [4], and 20% of female and 6% of male students [1] have experienced sexual violence while in college [1,2]. Sexual violence, defined by the Centers for Disease Control and Prevention as “a sexual act that is committed or attempted by another person without freely given consent of the victim or against someone who is unable to consent or refuse,” includes sexual assault, rape, and sexual coercion [5]. Although most commonly perpetrated by individuals known to the victim or survivor, including and oftentimes an intimate partner, sexual violence also includes unwanted acts used by persons who are not intimate partners and by persons not known to the victim or survivor [5].

IPV and sexual violence have been associated with increased risk of anxiety and depression, suicidal ideation, migraines, unprotected sex, unintended pregnancy, reduced access to reproductive health services, alcohol and substance use, and HIV and other sexually transmitted infections [2,6]. Compared with students unexposed to violence, college survivors of sexual assault are significantly more likely to have reduced grade point averages and slower time to completion of their degree and have an increased likelihood of leaving college or university altogether [7].

Data from 71,421 undergraduates found higher odds of sexual assault among cisgender women (vs cisgender men), transgender people (vs cisgender men), gay (vs heterosexual) men, and bisexual (vs heterosexual) students [8]. A study at a Hispanic-serving institution found that sexual and gender minority undergraduate students who experienced past-year violence were more than twice as likely to report some type of interference with their academic lives (eg, obtaining poor grades and missing class or work) compared with heterosexual, cisgender students who experienced past-year violence [9]. Studies have consistently found that violence is perpetrated at higher rates against students with (vs without) disabilities both during [10-12] and before enrolling in college or university [12]. This body of research highlights the need for culturally, racially, socially, and gender-relevant services for survivors of sexual and relationship violence.

### White House Task Force to Protect Students From Sexual Assault

In January 2014, President Barack Obama established the *White House Task Force to Protect Students from Sexual Assault* (hereinafter referred to as the *White House Task Force*) to strengthen federal enforcement efforts and provide recommendations and tools that colleges and universities could use to address sexual assault on their campuses [13]. Since its establishment, US institutions of higher education have increasingly adopted approaches to address campus-based violence. Many schools have received funding through the Office on Violence Against Women *Campus Program* of the US Department of Justice, created by Congress to provide grants to develop and strengthen trauma-informed victim services and strategies to prevent, investigate, and respond to sexual assault, sexual harassment, domestic violence, dating violence, and stalking [14]. Other schools have used institutional funding to establish and support violence prevention programs, including the University of California (UC), a public university system of 10 campuses that identifies preventing and responding to sexual violence and sexual harassment (SVSH) as top priorities.

### UC Sexual Assault Prevention and Response

In June 2014, in response to the *White House Task Force*, UC President Janet Napolitano formed the *President’s Task Force on Preventing and Responding to Sexual Violence and Sexual Assault* to improve current UC sexual violence prevention processes and develop recommendations for implementing strategies to improve prevention, response, and reporting procedures [15].

Between June 2014 and January 2016, UC implemented 7 components of an intended *comprehensive* system-wide model for addressing campus SVSH. These included (1) creation of a system-wide website for access to campus resources and important information; (2) mandatory education and training on sexual violence issues and prevention; (3) establishing a *Campus Assault Resources and Education (CARE): Advocate Office for Sexual and Gender-Based Violence and Sexual Misconduct* on each campus; (4) designating individuals on each campus to help respondents (ie, perpetrators) understand their rights and the investigation and adjudication processes of UC; (5) strengthening UC policy against sexual and domestic violence, stalking, and harassment as part of ongoing compliance with the federal Violence Against Women Act; (6) following a standardized 2-team response model at each campus (including 1 team for case management to review sexual misconduct reports and a second team focused on policies, community relations, prevention, and intervention using a campus collaborative approach); and (7) system-wide procedures for investigating, adjudicating, and imposing sanctions in student cases of SVSH [15].

Although the multicomponent approach of UC to address SVSH has established a strong foundation for cultivating a system-wide culture of respect and safety, it is not *comprehensive* per the definition used by the *White House Task Force*. Their recommended model for comprehensively assessing and responding to campus violence includes four action steps: (1) identifying the prevalence and determinants of sexual assault on campus through climate surveys, (2) developing evidence-based prevention strategies for sexual assault, (3) establishing investigation and adjudication procedures to respond to reports of sexual violence, and (4) improving federal enforcement efforts [13]. Missing from the approach of UC is the implementation of a campus climate survey and the development of evidence-based prevention strategies.

Campus climate surveys have been recommended for assessing the scope and context of violence on campuses to create and maintain effective, relevant violence prevention and response programs that are acceptable to the students and meet the needs of student survivors [13,16]. To date, UC Berkeley is the only UC campus that has conducted a climate survey focused on sexual violence and other forms of sexual, dating, and relationship harm. Thus, we lack evidence on the scope and nature of SVSH across the UC system, precluding our ability to tailor programs to meet the needs of each campus population.

### Objectives

This paper describes the protocol for phase 1 (2017-2021) and phase 2 (2020-ongoing) of a mixed methods research study conducted on 3 UC campuses. The goal of this research is to prepare for future implementation of a quantitative climate survey or an alternative research design that will allow for systematic, in-depth assessment of the prevalence, determinants, and nature of campus-based violence. Textbox 1 shows the 6 research aims of this study.

The design of this project was informed by guidelines from the comprehensive campus sexual assault climate assessment model developed by the Center on Violence Against Women and Children (VAWC) of Rutgers University [17,18] and from the implementation overview and lessons learned report of the MyVoice Working Group [19].

**Research aims of this study.**

**Research aims**

- Aim 1: assess students’ perceptions of sexual consent
- Aim 2: understand students’ perceptions of the campus environment related to sexual assault, sexual harassment, and dating violence
- Aim 3: investigate institutional and community arrangements influencing students’ lives and experiences
- Aim 4: examine how campus prevention, education, and response efforts can be tailored to meet the unique needs of diverse individuals and communities
- Aim 5: learn about student survivors’ use and perceptions of campus- and community-based violence and mental health services
- Aim 6: lay the groundwork for subsequent quantitative research and effective prevention programs coupled with healing-centered comprehensive response services at each campus

## Methods

### Study Setting and Timeline

**Figure 1:**
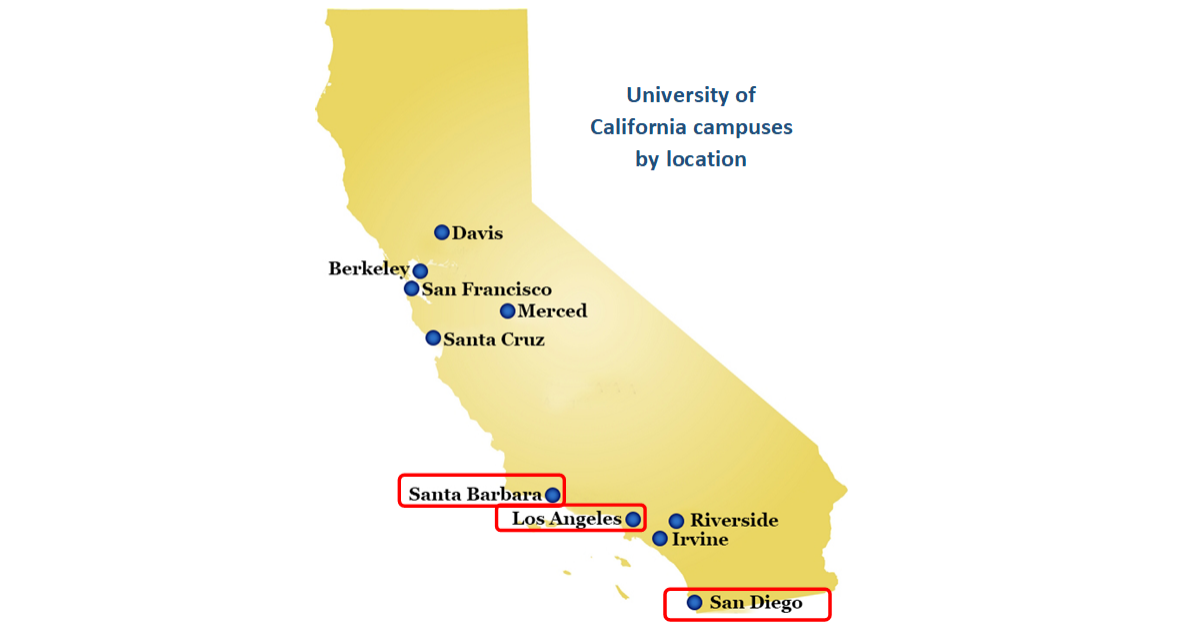
Map of California showing the 10 University of California campuses and highlighting the 3 campuses involved in this study.

**Figure 2:**
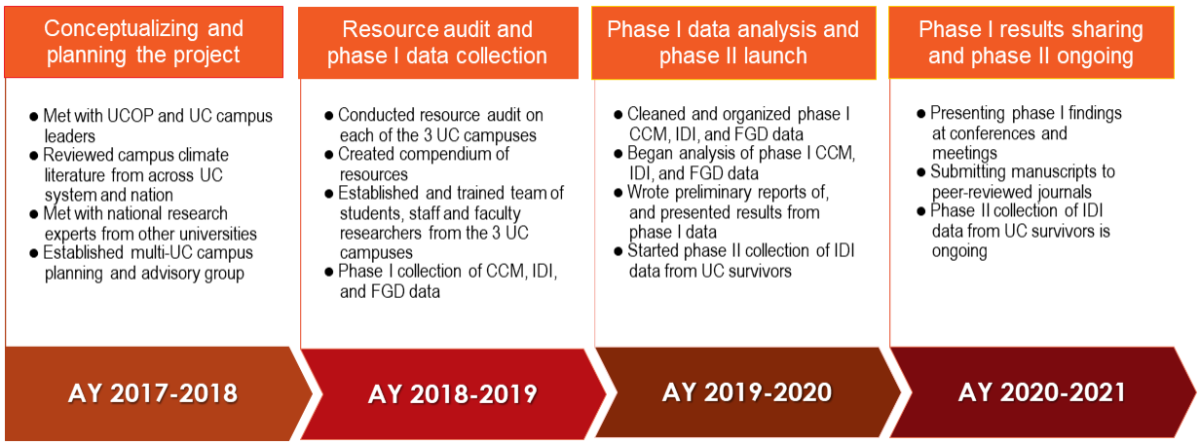
Timeline and steps of planning and implementing the project from academic year (AY) 2017-2018 to AY 2020-2021. CCM: cultural consensus modeling; FGD: focus group discussion; IDI: in-depth interview; UC: University of California; UCOP: University of California Office of the President.

There are 10 campuses in the UC system, and this project was conducted on three in Southern California: UC Los Angeles (UCLA), UC San Diego (UCSD), and UC Santa Barbara (UCSB; Figure 1). The project began during the 2017-2018 academic year (AY). The main phase of data collection was conducted in AY 2018-2019, during which time student enrollment by campus was as follows: 30,873 undergraduate and 14,074 graduate students at UCLA; 30,285 undergraduate and 8513 graduate students at UCSD; and 23,070 undergraduate and 2906 graduate students at UCSB [20-22]. The full project timeline is shown in Figure 2.

### Conceptualizing and Planning the Project (AY 2017-2018)

Project conceptualization began with a visioning and prioritization workshop at a meeting of faculty, staff, and students involved with the Women’s Health, Gender, and Empowerment Center of Expertise (WHGE-COE) of the UC Global Health Institute. WHGE-COE participants from all 10 UC campuses identified prevention of campus-based sexual violence as a high priority for system-wide mobilization and collaboration. Official project planning began by reviewing the literature on campus-based violence prevention research from UC and other US colleges and universities. Concurrently, we began iteratively reaching out to, meeting with, and gathering input from leaders and key stakeholders. All planning and advisory group participants and stakeholders are listed in Textbox 2.

To learn from experts in campus-based violence prevention research, we invited leaders from 4 experienced teams to consult with our group. A total of 2 half-day learning sessions were led by the Center on VAWC of Rutgers University (drawing on experiences with the #iSPEAK Campus Climate Survey) and the PATH to Care Center at UC Berkeley (drawing on experiences with the MyVoice Survey). In total, 2 full-day consultations were led by researchers from Columbia University (drawing on experiences from the Sexual Health Initiative to Foster Transformation study) [18] and from the Division of Student Life of the University of Oregon (drawing on their Crisis Intervention and Sexual Violence Support Services). Participants included the faculty leads from each campus; the WHGE-COE Directors; the UCSD Center on Gender Equity and Health staff research associate; the UC Office of the President team; and the CARE Directors from the UC Irvine, and UCSD Advocate Offices for Sexual and Gender-Based Violence and Sexual Misconduct.

**Project conceptualization, planning, and advisory group members.**

**Campus, office, or center and position**

**University of California (UC), Berkeley**

- Violence prevention counselors and advocates from the PATH to Care Center at UC Berkeley
- MyVoice survey research team members

**UC Irvine**

- Director of UC Irvine Campus Assault Resources and Education office

**UC Los Angeles**

- Associate Professor of Psychiatry and Biobehavioral Sciences, and Epidemiology (these 2 positions are held by the same person)
- Doctoral student of Community Health Sciences

**UC San Diego (UCSD)**

- Assistant Professor of Medicine
- Graduate student ambassador to Women’s Health, Gender, and Empowerment Center of Expertise (WHGE-COE)
- Director of UCSD Campus Assault Resources and Education office

**UC Santa Barbara**

- Professor and Chair of Feminist Studies
- Undergraduate student ambassador to WHGE-COE

**UC Office of the President**

- Vice President of Student Affairs
- System-wide Title IX director
- System-wide Title IX coordinator

**WHGE-COE**

- Codirector from UC Berkeley
- Codirector and UC Los Angeles Associate Professor (these 2 positions are held by the same person)
- Deputy Director of Research from UC San Francisco
- Deputy Director of Education from UC Santa Barbara
- Deputy Director of Violence Prevention Research and UCSD Assistant Professor (these 2 positions are held by the same person)

**UCSD Center on Gender Equity and Health**

- Codirector and Professor of Medicine
- Staff research associate
- Doctoral fellow

### Resource Audit and Phase I Data Collection (AY 2018-2019)

#### Resource Audit

We conducted a resource audit on each campus to examine available information on responding to and preventing IPV and sexual violence, gather input from key informants and stakeholders, and develop relationships with campus community members. This process was informed by the guidelines of the Center on VAWC of Rutgers University [23]. The assessment was coordinated by the study’s faculty investigators at UCLA, UCSD, and UCSB, who led all activities with assistance from their teams and from undergraduate and graduate WHGE-COE student ambassadors on each campus. The resource audit involved 3 main steps.

First, we gathered information through web-based searches, phone calls, and in-person office visits. This was done to explore UC-wide and campus-specific SVSH policies, investigative and adjudicative protocols, campus- and community-based support services for student sexual assault survivors, and on-campus prevention programs to reduce sexual and relationship violence.

Second, we engaged key stakeholders. At each campus, we spoke with the CARE Director or an advocate from the CARE office, the Title IX coordinator or a Title IX officer, a residential housing administrator, providers from student health services and Counseling and Psychological Services, the Director of Student Affairs, a director or administrator from athletics, and representatives from the Office of Equity, Diversity, and Inclusion; campus police or security; the campus Panhellenic Council; the International Student Center; the LGBT Resource Center; the Undocumented Student Center; and the Black and African American Student Center. Each stakeholder was invited to review and provide feedback on the list of resources gathered, suggest others we should talk with, and make recommendations for the study.

Third, information gathered during steps 1 and 2 was used to compile a compendium of resources for each campus. The findings were also used to tailor study design, decide on research methods, and inform the development of research questions. Contacts made during the audit contributed to long-term partnerships and introduced us to people who became members of the research team.

#### Establishing and Training the Research Team and Branding the Project (November-December 2018)

The full research team was established in November 2019 and included 6 faculty investigators, 3 staff coordinators, and 16 student investigators (10/16, 63% undergraduate and 6/16, 38% graduate students) divided evenly across the 3 UC campuses. In December 2019, all team members participated in a 3-day, in-person training at the UCSD School of Medicine campus. Training focused on (1) research ethics and how to conduct safe and trauma-informed research on SVSH; (2) how to provide short-term mechanisms of support to any participant triggered or distressed by the topics addressed in the study; (3) how to practice *self-care* given the potentially traumatic nature of the research; and (4) where to refer participants for additional, comprehensive services on each UC campus. Through participatory discussion, we named our study the UC Speaks Up Project and decided on the following guiding values: student-centered, evidence-based, health-centered, intersectional, inclusive, trauma-informed, and ethical.

#### Data Collection Procedures by Method and Phase

UC Speaks Up uses three research methodologies: cultural consensus modeling (CCM), qualitative in-depth interviews (IDIs), and focus group discussions (FGDs). The first and main phase of data collection was conducted between January 2019 and June 2019. All 3 methodologies were used, and data were gathered from students, staff, and faculty from the 3 UC campuses and local stakeholders from communities surrounding each campus. Phase II of data collection, a smaller subinvestigation with sexual violence survivors, began in September 2020 and is ongoing. The methods are described in detail below by phase.

#### CCM With Students (Phase I: January 2019)

##### Overview

CCM is a technique for estimating the extent to which people share common beliefs and understandings about a topic. Individuals who answer questions about their culture in a similar pattern are assumed to be giving the most culturally salient or correct answer [24,25]. We used CCM to understand (1) if there was a culture of sexual consent on campus (in other words, if students had a common frame of reference for consent that they could reasonably expect their partners to share), (2) if that culture of consent varied by gender or other demographics, and (3) what knowledge (rules) constituted the culture of consent. CCM allowed us to identify *answer keys* of the culturally correct meaning of sexual consent by identifying clusters of similar informant responses. It also allowed us to identify cultural experts; that is, individuals who provided a large number of *culturally salient or correct* answers (according to the identified answer key) and were presumed to likely have a large amount of expertise on the topic. The CCM process was performed in 3 steps using 2 types of data collection (free-listing interviews and a web-based survey).

##### CCM Step 1: Free-Listing CCM Interviews

The CCM process began with the use of free listing, a technique for gathering data about a specific cognitive domain by asking people to list all the items they can think of that fall into that category. At each campus, student researchers approached fellow students in public spaces, such as the quad or the library, introduced themselves, explained the purpose of the study, and invited them to participate. Interested students were asked to disclose their age, gender identity, and student status (undergraduate, graduate, or professional student). Eligibility criteria included self-reporting as a current student of the UC campus where data collection was being conducted, being between the ages of 18 and 26 years, and providing verbal consent. After obtaining verbal consent, student participants were asked the following three questions*:* (1) *How do students in your campus community know their partner is signaling sexual consent?* (2) *How do students in your campus community signal their own sexual consent?* (3) *What words would students in your campus community use to describe a sexual encounter that feels good?* Up to 10 responses for each question were recorded by the researcher. Drinks and candy bars were provided to all the participants.

##### CCM Step 2: CCM Survey Development

We analyzed the free-listing responses to the 3 CCM questions using Microsoft Excel and the software package *AnthroTools* (R Foundation for Statistical Computing) [26] to create the web-based survey. We reviewed all responses to each question, then tallied the number of *unique responses* to each question (ie, if 3 students provided the same response for 1 question, 1 item reflecting that response was included in the list of possible items). Item salience was calculated using Smith *S* scores to rank averages across all samples separately by gender. Items were weighted by the order in which they were provided. The average Smith *S* score was ranked to determine the top 20-30 items by gender. The Smith *S* score determines the item’s salience based on both the frequency with which participants mention a free-list item as well as the order in which an item is mentioned. If a free-list item is the first or second response given by a large number of participants, it is considered to be highly salient. The Smith *S* is calculated as follows:

S = ((∑(L – R_j_ + 1))/L/N)

where *L* is the number of items on the list (length), *R* is the order in which item *j* is mentioned (rank), and *N* is the number of items on the list [27].

We then created a web-based survey in REDCap (Vanderbilt University) by including items with high Smith *S* scores as well as some items of interest (based on our literature review and resource audit) with lower salience, such as *sober* and *not resisting*.

##### CCM Step 3: Web-Based Survey

Survey participants were recruited using a convenience sample approach across all 3 campuses. The study was advertised via email and social media. We printed paper flyers that were displayed in public spaces on campus. All recruitment materials provided information about the study, details of participation, contact information for the investigators, and a link to follow to be screened for eligibility. Eligibility criteria included being a current student at UCLA, UCSD, or UCSB; being aged 18-26 years; and providing electronic informed consent. Although these criteria likely excluded some re-entry, graduate, and professional students, the age range eligibility was restricted as individuals aged >26 years were more likely to have different generational life-course understandings of consent than participants aged 18-26 years. A 2-step recruitment and enrollment process was used. First, to ensure enrollment at UCLA, UCSD, or UCSB, students were required to enter their campus email address. Second, students with authentic UCLA, UCSD, and UCSB email addresses were sent a unique link to access the web-based survey. Clicking the link brought the participant to a page with a complete consent form and contact information for the principal investigator and research contact person. After reading the form, the participants were prompted to provide their digital signature if they consented to participate, which would enable them to proceed to the survey. Participants were enrolled until the target sample size of 250 was achieved. This sample size was a conservative target, based on recommendations to assume a low level of agreement (such as 50%) and require high validity (such as 0.95%) when beginning a new study [24]. Drawing on Weller’s [24] sample size and validity estimates for different levels of agreement, we assumed a sample size of 250 would allow us to detect a significant consensus model with 99% validity, even with a low-average level cultural competency score of 0.40, which is equivalent to an average 0.16 Pearson correlation coefficient between respondents, based on Weller’s [24] estimates.

All participants were given a US $5 electronic gift card as compensation for their time.

The web-based instrument collected demographic information—campus, age, gender identity, sexual orientation, level in school, field or discipline or major, residency and housing status, and group membership (eg, part of sports or athletics or student government)—and asked the participants to rate the importance of items identified during the free-listing phase. For each domain (*How do students in your campus community know their partner is signaling sexual consent?* *How do students in your campus community signal their own sexual consent?* *What words would students in your campus community use to describe a sexual encounter that feels good?*), the participants were presented with approximately 20 items derived from the free-listing phase and asked to rate the items from 1 to 7 to describe how important that item was as a strategy to recognize consent or signal consent or as a way of describing a positive sexual encounter. The participants were prompted to rank the level of importance of 24 options for question 1, 28 options for question 2, and 21 options for question 3. The complete survey is provided in Multimedia Appendix 1.

#### IDI and FGD Methodology

##### Recruitment of IDI and FGD Participants

Related to phase I participant recruitment, additional eligibility criteria for students included self-reported enrollment in an undergraduate, graduate, or professional program at UCLA, UCSD, or UCSB. UC faculty and staff were only eligible if they were currently employed by the UC and had been in that position for at least 6 months. Additional eligibility criteria for community stakeholders were currently working at a sexual violence–related, sexual harassment–related, or domestic violence–related service agency; having been in that position for at least 6 months; and having experience in helping students seeking violence-related services or support within the Los Angeles, San Diego, or Santa Barbara region. A subset of eligible UC students, staff, and faculty participants was selected based on key demographics (eg, gender identity, sexual orientation, race and ethnicity, year in program, type of program, and academic department) to attempt to achieve representation at the group (ie, student, staff, and faculty) and campus level. Related to phase II participant recruitment, additional eligibility criteria for student survivors included being currently enrolled at one of the 3 UC campuses or having graduated within the last 3 years and self-reporting experience of sexual assault, sexual harassment, stalking, or dating violence while enrolled as a UC student. Participants selected for inclusion were connected with a trained UC Speaks Up student, staff, or faculty researcher to schedule an IDI or FGD.

##### Structure of IDIs and FGDs and Compensation of Participants

IDI and FGD data were gathered using semistructured guides with open-ended questions that allowed for conversational inquiry on the research topics described above. Probes were used to elicit additional information or clarify responses. Phase I data collection occurred on campus in accessible and convenient locations where privacy could be ensured. In-person IDIs and FGDs during phase I were audio-recorded, and the participants received a US $25 Visa gift card in compensation for their time. Remote interviews with survivors during phase II have been conducted via the Zoom platform on a day and at a time agreed upon by both the researcher and participant. Participants in remote interviews are invited to use both audio and video features during the interviews but are assured that the video is voluntary. Remote IDI participants receive a US $50 electronic gift card in compensation for their time. Compensation is higher in phase II than in phase I as we estimated that (1) the interviews might last longer and (2) the interviews may be more taxing owing to the highly sensitive nature of sexual violence and the potential for increased risk of participants feeling distressed or triggered by discussing past experiences. All IDI and FGD participants throughout the study are provided with a resource sheet unique to their campus with comprehensive details of on-campus and community-based services (based on information collected during the resource audit).

#### Phase I (February 2019-June 2019) IDIs With Students, Staff, Faculty, and Community Stakeholders

IDIs were conducted with students, staff, faculty, and community stakeholders. IDIs with students aimed to explore their attitudes about relationships and sex; their definitions of sexual violence, sexual harassment, and healthy relationships; and their awareness of available services, prevention programs, and policies addressing sexual violence at the university. We sought students’ opinions on how they can become more involved in making the campus an environment that does not tolerate sexual or gender-based violence. IDIs with faculty, staff members, and university administrators examined how they perceive their role and their office’s role in prevention, education, and response services addressing sexual violence. The IDIs also aimed to learn about the process they and their office take when a student discloses. IDIs with community stakeholders were structured to explore their relationship with their university counterparts and assess the services and programs they offer to UC students and the larger community. The interviews lasted, on average, between 60 and 90 minutes (SD 15 minutes).

#### Phase I (April 2019-June 2019) FGDs With Students

FGDs were conducted with students and aimed to understand group norms surrounding the campus environment and how students felt about campus safety, healthy socializing, and acceptance and rejection of relationship violence. We explored students’ definitions of healthy versus unhealthy relationships and sex as well as sexual assault and sexual harassment. Each discussion was facilitated by a trained moderator and note-taker. FGDs allowed for discussion of general themes, including awareness of services and education activities, challenges in accessing care and services, and ideas for prevention messaging that resonated with them. FGDs lasted, on average, 90 (SD 30) minutes.

### Phase I Data Analysis and Phase II Launch (AY 2019-2020)

#### Analysis

##### Overview

In September 2019, we started analyzing the phase I data. This process, together with data interpretation, report, and manuscript development and results dissemination (through workshops, meetings, and conferences), is ongoing. Analysis of phase II data with survivors has not yet started. Descriptive analyses have been or will be conducted for demographic variables gathered for all the participants. Simple frequency distribution statistics (eg, mean and proportion) will be conducted using Stata (version 15.1; StataCorp).

##### Aim 1: Assess Students’ Perceptions of Sexual Consent

Aim 1 involved the analysis of CCM survey data, which has been completed. Interview and focus group discussion data analysis is also part of aim 1, some of which has been completed and the rest is ongoing.

CCM survey data were entered into R software (R Foundation for Statistical Computing) [28] and analyzed by gender, age, and housing status using AnthroTools to determine (1) whether there were clusters of similar item ratings (ie, cultural consensus models) either across the full group of students or by gender, age, or housing status and, if so, (2) what the culturally *correct* rating or importance of each item was. Consistent with the methods by Weller [24], we considered a cluster of similar answer ratings to represent a distinct cultural consensus model if the group’s eigenvalue was >3.0.

All qualitative IDI and FGD data have been or will be analyzed using Dedoose (SocioCultural Research Consultants, LLC [29]), a mixed methods web-based analysis platform. Qualitative content analysis is used to generate substantive codes and subthemes that emerged from the data for all domains we are examining. Primary domains are predetermined based on the semistructured interview and focus group guides, and subtheme code identification was or will be informed using a thematic analysis approach. A coding tree was or will be developed by the team for each aim after iterative rounds of discussion around substantive codes that evolved into tangible themes. The codes produced were or will be organized into broad conceptual codes (ie, parent codes) and more refined subcodes (ie, child codes). Discrepancies in codes are resolved through group discussion. At least 2 reviewers coded each transcript to ensure interrater reliability.

##### Aim 2: Understand Students’ Perceptions of the Campus Environment Related to Sexual Assault, Sexual Harassment, and Dating Violence

We analyze IDI and FGD data from student participants, starting with an exploration of students’ definitions of healthy versus unhealthy relationships, sexual assault, sexual harassment, stalking, and dating violence, to achieve this aim. FGD data are examined to understand group norms surrounding the campus environment for safety, opportunities for healthy socializing, and sexual and relationship violence. All data are analyzed to assess perceptions of whether violence is a problem on campus, how students think the university handles and responds to violence, and what are the levels of awareness about sexual violence services and programs. Data from IDIs and FGDs with graduate students further examine how power relations with faculty and trust or distrust of university processes contribute to graduate students’ decisions about seeking services. The data capture graduate students’ recommendations for improving campus climate and SVSH resources to meet graduate students’ needs.

##### Aim 3: Investigate Institutional and Community Arrangements Influencing Students’ Lives and Experiences

To achieve this aim, we analyze IDI data from staff, faculty, and community stakeholders to gain a full picture of the services, protocols, and policies related to violence that are available on the campuses and surrounding communities. We will assess how faculty and key university administrators perceive their roles in supporting survivors who disclose abuse, harassment, or discrimination or who want to report an incident to a professional, such as a Title IX coordinator or law enforcement officer. We aim to learn how UC faculty and staff perceive their preparedness to contribute to both prevention and response efforts and where they feel gaps remain so recommendations can be made on where additional training and support is required. Another key component of this aim is the analysis of data from community stakeholders to assess relationships between local violence prevention advocates and the UC and explore how these relationships can be strengthened to improve prevention of sexual violence in and around each campus.

##### Aim 4: Examine How Campus Prevention, Education, and Response Efforts Can Be Tailored to Meet the Unique Needs of Diverse Individuals and Communities

In response to the literature suggesting that students from racial, ethnic, gender, and sexual minority populations as well as students with disabilities are disproportionately burdened by SVSH [8-12], we will analyze IDI and FGD data from students, staff, and faculty to discern the needs and preferences regarding SVSH prevention and response among both the general population and historically marginalized groups. We explore unique cultural and contextual configurations that emerge in conversations about SVSH in these populations. We also assess potential SVSH-related stressors associated with unique identities and barriers to accessing or continuing the use of physical and mental health, psychosocial, and other SVSH services. These findings will facilitate the development of tailored programs for subgroups.

##### Aim 5: Learn About Student Survivors’ Use and Perceptions of Campus- and Community-Based Violence and Mental Health Services

In-progress interviews with survivors will be transcribed, coded (as described above), and analyzed to explore survivors’ experiences of SVSH while enrolled as UC students. Codes will be developed to assess the number, frequency, and types of and overlap between different forms of harm and to examine what impact these experiences had on survivors’ lives. We will examine what actions survivors took after the incident or incidents, including disclosure, use of services, legal actions, and pursuit of criminal justice, and what their perceptions were of these experiences and interactions. Recommendations provided by survivors will be recorded and distributed to service providers and administrators. The findings will be assessed overall, by campus, and by specific subgroups (race, ethnicity, gender orientation and sexual identity).

##### Aim 6: Lay the Groundwork for Subsequent Quantitative Research and Effective Prevention Programs Coupled With Healing-Centered Comprehensive Response Services at Each Campus

Building on the findings from the first phase of UC Speaks Up, we launched the *Listening to UC Survivors Study*, which aims to interview student survivors of SVSH to create recommendations on how the UC response and prevention systems can be improved to create a safer learning environment. In addition, 3 student-led qualitative research projects have been launched or are under development. *Double Jeopardy: Asian International Students*’ *Experiences of Sexual Violence and Xenophobia during COVID-19* explores Asian international students’ experiences of SVSH during their time in the United States both before and during the COVID-19 pandemic. This study has received institutional review board (IRB) approval and is in the data collection phase. A second study (under development) aims to address lesbian, gay, bisexual, transgender, and queer students’ unique needs in relation to SVSH. A third study (also under development) addresses perceptions of how the COVID-19 pandemic has affected SVSH within the Greek community (fraternities and sororities) at UCLA. These 3 student-led studies will be described in detail elsewhere (ie, not in this paper).

#### Launch of Phase II: IDIs With Student Survivors (September 2020-Ongoing)

We are conducting IDIs with current and recently graduated (ie, within the past 3 years) students who experienced sexual assault, sexual harassment, or dating violence while enrolled at UCLA, UCSD, or UCSB. We plan to conduct approximately 30 IDIs with 10 survivors from each campus. These interviews aim to learn what services and programs student survivors use on their UC campus or in the surrounding community; hear their perspectives on what was most or least helpful when dealing with experiences of violence; and seek recommendations for how the UC system can improve in terms of both preventing and responding to violence, harassment, and discrimination.

### Phase I Results Sharing and Phase II Ongoing (2020-Ongoing)

#### Phase I Results Sharing

The findings from phase I have been and continue to be presented at professional and academic conferences across the globe. Student-led subanalyses of phase I data include barriers to access to care, SVSH among historically marginalized communities, student-generated recommendations to improve universities’ responses to SVSH, student athletes’ perceptions of SVSH, and the relationship between alcohol consumption and SVSH. To date, 5 academic papers exploring results from UC Speaks Up have been either published or accepted for publication [30-34].

#### Phase II Ongoing

To date, 15 interviews have been completed with student survivors of SVSH. Recruitment will continue throughout the 2021-2022 AY. We planned to begin these interviews in March 2020 after completing the analysis of phase I data. However, owing to the COVID-19 pandemic, we did not start until September 2020 because of the need to revise our research protocol—from in-person to remote IDIs—and receive IRB clearance. We took a 6-month break from data collection between March and August 2021 owing to hardship related to the ongoing COVID-19 pandemic and resumed interviews in September 2021. All the phase II survivor interviews are being conducted via the web-based teleconferencing software Zoom using a secure link and password-protected meeting space.

### Data Management and Quality Assurance

All interviews and FGDs were or will be transcribed verbatim from audio recordings either directly into a Word document or using the transcription platform, Trint [35]. Transcripts were or will be redacted to remove personal identifying information and stored in a shared, encrypted file. All data files were or will be reviewed and cleaned (as needed) by a data manager to ensure they are properly labeled and complete. If details were or are missing from a file (eg, a participant’s demographics), the data manager tried or will try to locate this information to complete the file.

Although the study procedures are minimally invasive and present low risks to the participants, we established numerous safeguards and followed several precautions to protect participants and ensure data confidentiality. The participants are assigned a numeric personal ID number that is used as a reference to the participant instead of their name on all study data. This number delinks personal identifying information from the study databases. The names of the participants are kept in separate secure files. All paper data collection tools are stored in secure, locked facilities at UCLA, and only a small number of designated staff members have access to these records. All electronic data are stored in encrypted, password-protected files that are only accessible to the study’s principal investigators.

### Safe and Ethical Conduct of Human Subjects Research Approval

The study protocol for phase I was approved by the UCSD Human Research Protection Program (HRPP) (Approval number: 181722). Agreements to rely on the UCSD HRPP were approved by the IRBs at UCLA (Approval number: 18-001885) and UCSB (Approval number: 128-19OA-1). In July 2019, the UC Speaks Up principal investigator (the first author) relocated from UCSD to UCLA and the study protocol for phase II was submitted to and approved by the UCLA HRPP (Approval number: 20-000445). Since all interviews for phase II are being done remotely by UCLA researchers, the IRBs at UCSD and UCSB indicated reliance agreements were not required. Before working with the UC Speaks Up study, all research staff received training on the safe and ethical conduct of research on violence against women based on recommendations developed for the World Health Organization Multi-Country Study on Women’s Health and Domestic Violence [36]. Staff also received training on the ethical conduct of human subject research, compliance, and data management via a collaborative institutional training initiative for biomedical research. Students who took part in the CCM free-listing provided verbal consent before participating. CCM survey participants consented on the web before starting the questionnaire. Phase I IDI and FGD participants provided written consent to take part in the data collection and have the session audio-recorded. Phase II IDI participants provided oral consent to take part in the data collection and have the session audio-recorded. A certificate of confidentiality was obtained from the National Institutes of Health to protect identifiable, sensitive research information from compulsory legal disclosure (eg, sexual assault).

## Results

### Free-Listing and Web-Based Survey Participants

Free-listing interviews were conducted with 149 students, and data were analyzed from 122 (81.9%) participants (input from 27/149, 18.1% of students was excluded for lack of data on age or because the participants were aged >26 years). Unique item responses were tallied for partners’ signals of consent (n=149), students’ own signals of consent (n=209), and students’ descriptions of a *good* sexual encounter (n=277). Most (119/149, 80%) of the students who participated were undergraduates, and 20% (30/149) were graduate or professional students. Ages ranged from 18 to 26 years, and the mean age was 21 (SD 2.4) years. Approximately 60% (84/149) identified as the female gender, and 40% (61/149) identified as the male gender.

Web-based surveys were completed by 214 students (177/214, 83% undergraduate and 37/214, 17% graduate and professional) from UCLA (43/214, 20%), UCSD (77/214, 36%), and UCSB (94/217, 44%). The participants identified their race and ethnicity as Asian (104/217, 42%), White (83/217, 33%), Latinx or Spanish or Hispanic (34/214, 14%), Black or African American (11/214, 4%), Native Hawaiian (5/214, 2%), and Indigenous or Native American (1/214, 1%). In terms of gender and sexual identity, 61% (131/214) identified as female, 38% (81/214) identified as male, and 1% (2/214) identified as nonbinary. Approximately 76% (163/214) identified as heterosexual or straight, and 24% (43/214) identified as lesbian; gay; bisexual; transgender; queer or questioning; intersex; asexual; and all other sexualities, sexes, and genders (LGBTQIA+).

No consensus was found among students with regard to their understanding of any type of sexual consent. We interpreted this finding to suggest that there is wide variation in students’ conceptions of what sexual consent is and how it is signaled by a partner. It also indicates that students may refrain from talking with their peers about how to signal consent or interpret their partner’s consent signals. We used these findings to inform the development of our IDI and FGD guides, to include questions to assess students’ lived experiences of making (or not making) agreements with partners to participate in a sexual activity, and to inquire about the process of setting personal boundaries and respecting those of a partner.

### Interview Participants Enrolled in Phase I

A total of 179 IDIs were conducted with 86 (48%) undergraduate students, 21 (11.7%) graduate and professional students, 34 (19%) staff and administrative members, 27 (15.1%) faculty members, and 11 (6.1%) community stakeholders (Table 1).

A total of 86 undergraduate student IDI participants were recruited from UCLA (26/86, 30%), UCSD (30/86, 35%), and UCSB (30/86, 35%) and included first- (21/86, 24%), second- (20/86, 23%), third- (20/86, 23%), fourth- (21/86, 24%), and fifth-year (4/86, 4.6%) students. The participants were drawn from majors in the humanities, social sciences, and arts (32/86, 37%) as well as science, technology, engineering, and math (54/86, 63%). Slightly more than half (47/86, 55%) identified as cisgender women; 39% (34/86) identified as cisgender men; and 6% (5/86) identified as agender, nonbinary, or transgender. Most (62/86, 72%) identified as heterosexual, 11% (9/86) identified as bisexual, 8% (7/86) identified as lesbian or gay, 4% (3/86) identified as pansexual, 3% (2/86) identified as nonconforming, and 3% (3/86) identified as asexual or mostly heterosexual. The participants identified as White (30/86, 35%), Asian (20/86, 23%), Latinx or Spanish or Hispanic (14/86, 16%), Black or African American (11/86, 12%), South Asian or Indian (3/86, 3%), Middle Eastern (4/86, 5%), and more than one race (4/86, 5%). Only 3% (2/86) of the participants reported living with a disability.

A total of 21 graduate and professional student IDI participants were recruited from UCLA (8/21, 38%), UCSD (6/21, 29%), and UCSB (7/21, 33%) and included students enrolled in master’s degree programs (8/21, 38%) and doctoral degree programs, including doctor of philosophy (9/21, 43%), doctor of medicine (3/21, 14%), and juris doctor (1/21, 5%). Graduate and professional students were from the fields of bioengineering, bioinformatics, biology, communications, economics, education, engineering, fine arts, law, materials, medicine, public health, and sociology. Most (13/21, 62%) identified as cisgender women, 28% (6/21) identified as cisgender men, 5% (1/21) identified as agender, and 5% (1/21) identified as nonbinary. Approximately 57% (12/21) identified as heterosexual, 24% (5/21) identified as bisexual, 9% (2/21) identified as lesbian or gay, 5% (1/21) identified as asexual, and 5% (1/21) identified as nonconforming. By race and ethnicity, the participants identified as White (9/21, 43%), Asian (7/21, 33%), Latinx or Spanish or Hispanic (3/21, 14%), Black or African American (1/21, 5%), and more than one race (1/21, 5%). Approximately 10% (2/21) were living with a disability.

A total of 34 staff members were recruited from UCLA (11/34, 32%), UCSD (13/34, 38%), and UCSB (10/34, 29%) and included health and well-being service providers (7/34, 21%); athletic department staff (8/34, 25%); and staff from student affairs (6/34, 18%), academic departments (3/34, 9%), and student resources (10/34, 29%). Health and well-being service providers included clinicians, therapists from Counseling and Psychological Services, and sexual violence service providers from CARE. Athletic department staff included directors, coaches, and administrators. Student affairs staff held positions such as Dean of Student Affairs and Student Life Development Specialist. Student resource staff held positions such as Director of the Undocumented Student Services Center. Most staff (23/34, 68%) identified as cisgender women, 26% (9/34) identified as cisgender men, and 6% (2/34) identified as nonbinary. Approximately 58% (20/34) identified as heterosexual, 21% (7/34) identified as lesbian or gay, 16% (5/34) identified as gender nonconforming, and 5% (2/34) identified as asexual. Staff identified as White (18/34, 52%), Asian (2/34, 5%), Latinx or Spanish or Hispanic (5/34, 16%), Black or African American (5/34, 16%), Middle Eastern (2/34, 5%), and more than one race (2/34, 5%). Approximately 11% (4/34) were living with a disability.

A total of 27 faculty members were recruited from UCLA (8/27, 30%), UCSD (9/27, 33%), and UCSB (10/27, 37%) and included people from public health (3/27, 12%); science, technology, engineering, and math (10/27, 38%); and the humanities, social sciences, and arts (14/27, 50%). A range of disciplines were represented, such as Epidemiology, Psychiatry, World Arts and Cultures, Asian American Studies, English, Biology, Pharmacy, Engineering, Literature, Cognitive Science, and Philosophy. Most of the faculty interviewed were full professors (18/27, 65%), followed by associate professors (6/27, 23%) and assistant professors (3/27, 12%). Most chose not to disclose their race and ethnicity, gender identity, and sexual orientation. Thus, we do not report these statistics for this group.

A total of 11 stakeholders were recruited from the communities surrounding UCLA (3/11, 27% of participants), UCSD (4/11, 36% of participants), and UCSB (4/11, 36% of participants). In Los Angeles, we interviewed stakeholders from the Rape Treatment Center at the UCLA Medical Center in Santa Monica, the Center for the Pacific Asian Family, and Peace Over Violence. In San Diego, we interviewed stakeholders from the Institute on Violence, Abuse, and Trauma; Alliance for Hope International; Love on a Leash; and the Center for Community Solutions. In Santa Barbara, we interviewed stakeholders from the office of the Santa Barbara District Attorney Victim-Witness Assistance Program, Stand Together to End Sexual Assault, Domestic Violence Solutions, and an independent trauma therapist who frequently serves students at UCSB.

**Table 1:** Number of in-depth interviews (IDIs) conducted, classified by University of California campus and participant type (N=179).

Participant type	Campus		
	UCLA^a^ (n=56)	UCSD^b^ (n=62)	UCSB^c^ (n=61)
Undergraduate students (n=86), n (%)	26 (30)	30 (35)	30 (35)
Graduate and professional students (n=21), n (%)	8 (38)	6 (29)	7 (33)
Staff (n=34), n (%)	11 (32)	13 (38)	10 (29)
Faculty (n=27), n (%)	8 (30)	9 (33)	10 (37)
Community stakeholders (n=11), n (%)	3 (27)	4 (36)	4 (36)

^a^UCLA: University of California, Los Angeles.

^b^UCSD: University of California, San Diego.

^c^UCSB: University of California, Santa Barbara.

### FGD Participants

A total of 35 FGDs (10/35, 29% at UCLA; 13/35, 37% at UCSD; and 12/35, 34% at UCSB) were conducted with 201 total participants. Of the 35 FGDs, 27 (77%) were completed with undergraduate students, and 8 (23%) were completed with graduate and professional students. Table 2 shows the breakdown by participant type of the FGDs conducted across the 3 campuses.

A total of 27 FGDs (27/35, 77%) were conducted with undergraduate students recruited from UCLA (8/27, 30%), UCSD (9/27, 33%), and UCSB (10/27, 37%). A total of 158 students were involved in these FGDs (36/158, 22.8% from UCLA; 61/158, 38.6% from UCSD; and 61/158, 38.6% from UCSB). Groups with members of sororities and fraternities, National Collegiate Athletic Association athletes, and engineering students were conducted separately by gender identity. Undergraduate *student leaders* included participants involved in student government (eg, the Undergraduate Students Association Council) and other campus-based leadership positions (eg, the Student Leadership Council). An undergraduate FGD was conducted on the UCLA campus with SVSH prevention leaders, including student interns from the CARE office, members of the Bruin Consent Coalition, and a Title IX policy special interest group. On average, the undergraduate student focus groups had 8 (SD 2) participants.

A total of 8 FGDs (8/35, 23%) were conducted with graduate and professional students recruited from UCLA (2/8, 25%), UCSD (4/8, 50%), and UCSB (2/8, 25%). A total of 43 students were involved in these FGDs (12/43, 28% from UCLA; 22/43, 51% from UCSD; and 9/43, 21% from UCSB). Groups conducted with FGD participants from the liberal arts included master’s- and doctoral-level students from the natural sciences, social sciences, arts, and humanities. Health profession students were drawn from graduate programs in medicine, nursing, dentistry, pharmacy, and public health. On average, the graduate and professional student FGDs had 6 (SD 2) participants.

**Table 2:** Number of focus group discussions (FGDs) conducted, classified by University of California campus and participant type (N=35).

Participant type		Campus		
		UCLA^a^ (n=10)	UCSD^b^ (n=13)	UCSB^c^ (n=12)
**Undergraduate students (n=27)**				
	Sorority and fraternity members (n=5), n (%)	1 (20)	2 (40)	2 (40)
	NCAA^d^ athletes (n=7), n (%)	2 (29)	3 (43)	2 (29)
	LGBTIA+^e^ students (n=3), n (%)	1 (33)	1 (33)	1 (33)
	Student leaders (n=3), n (%)	1 (33)	1 (33)	1 (33)
	Black students (n=2), n (%)	0 (0)	1 (50)	1 (50)
	Latinx students (n=2), n (%)	0 (0)	1 (50)	1 (50)
	Engineering students (n=2), n (%)	0 (0)	0 (0)	2 (100)
	SVSH^f^ prevention leaders (n=2), n (%)	2 (100)	0 (0)	0 (0)
	General population (n=1), n (%)	1 (100)	0 (0)	0 (0)
**Graduate and professional students (n=8)**				
	LGBTIA+ students (n=2), n (%)	0 (0)	1 (50)	1 (50)
	Liberal arts students (n=2), n (%)	1 (50)	1 (50)	0 (0)
	Health profession students (n=1), n (%)	0 (0)	1 (100)	0 (0)
	Male graduate students (n=1), n (%)	0 (0)	0 (0)	1 (100)
	STEM^g^ students (n=2), n (%)	1 (50)	1 (50)	0 (0)

^a^UCLA: University of California, Los Angeles.

^b^UCSD: University of California, San Diego.

^c^UCSB: University of California, Santa Barbara.

^d^NCAA: National Collegiate Athletic Association.

^e^LGBTIA+: lesbian; gay; bisexual; transgender; intersex; asexual; and all other sexualities, sexes, and genders.

^f^SVSH: sexual violence and sexual harassment.

^g^STEM: science, technology, engineering, and math.

### Student Survivor Interview Participants Enrolled in Phase II (Ongoing)

To date, 15 participants have been enrolled and interviewed from UCLA (5/10, 50%), UCSD (5/10, 50%), and UCSB (5/10, 50%). Recruitment is ongoing. However, as the COVID-19 pandemic shifted the climate in which survivors experience and respond to sexual and other forms of relationship misconduct, we made revisions to our research materials (requiring additional IRB approvals and delays) to be more salient to survivors’ current lived experiences.

## Discussion

IPV and sexual violence remain important public health and social justice issues on college and university campuses across the United States. The mission of the UC Speaks Up research is to understand the factors shaping intimate relationships and sexual and interpersonal violence among students at UCLA, UCSD, and UCSB and use the findings to develop and test prevention and response interventions (including policy updates) to improve the health, safety, and well-being of all members of the UCLA, UCSD, and UCSB communities. Access to evidence from each campus will leverage our ability to make specific recommendations for tailoring response systems (eg, advocacy offices for survivors) and primary prevention approaches to ensure they are relevant and acceptable to and meet the needs of all individuals in the campus community, including those who are typically understudied.

The findings will also be used to prepare for rigorous public health prevention research on SVSH across the entire UC system. As the most comprehensive and advanced postsecondary educational system in the world [17], representative survey research is warranted across all 10 UC campuses. However, only 1 UC campus has previously conducted a focused SVSH climate study. We hope this protocol paper and our preliminary results as well as the findings from the UC Speaks Up research will build on the research tools of the MyVoice Working Group [19] and highlight the significant need for implementation of additional SVSH prevention research across the UC system.

We acknowledge several limitations inherent to this project. First, owing to purposive sampling, the samples might not be representative of each campus population. Therefore, our results might not be generalizable to the larger UC population or to other universities (eg, private schools and schools with smaller populations and in more rural settings). Second, some of our study’s participant groups (such as undocumented students, male athletes, and fraternity members) were difficult to reach and are not as represented in the sample even with increased efforts using snowball sampling. Thus, their perspectives may not be fully reflected in our findings, and we recommend that future studies consider oversampling these and other hard-to-reach populations to ensure that their unique perspectives are included. The COVID-19 pandemic also introduced a substantial challenge to our research plan and essentially stopped our data collection for the UC Survivors Study. We have tried to compensate for this gap in study flow by resuming fieldwork during the 2021-2022 AY and adapting our research instruments to assess the impact of the pandemic on SVSH.

Notwithstanding the limitations of our ongoing research, we feel the UC Speaks Up Project has and continues to increase our understanding of how we can better prevent and respond to sexual violence and misconduct on the UC campuses as well as at other institutions of higher education in the United States. The COVID-19 pandemic creates new challenges pertaining to social dynamics on college and university campuses, and epidemiological and social science research is currently more important than ever to ensure that SVSH prevention programs and support services can be tailored to meet the changing needs of survivors and their allies.

## Appendix

Multimedia Appendix 1 The Web-Based Cultural Consensus Model Survey.

## Abbreviations

AY: academic year
CARE: Campus Assault Resources and Education
CCM: cultural consensus modeling
FGD: focus group discussion
HRPP: Human Research Protection Program
IDI: in-depth interview
IPV: intimate partner violence
IRB: institutional review board
LGBTQIA+: lesbian; gay; bisexual; transgender; queer or questioning; intersex; asexual; and all other sexualities, sexes, and genders
SVSH: sexual violence and sexual harassment
UC: University of California
UCLA: University of California, Los Angeles
UCSB: University of California, Santa Barbara
UCSD: University of California, San Diego
VAWC: violence against women and children
WHGE-COE: Women’s Health, Gender, and Empowerment Center of Expertise
