# Supplementary material for: Student, Staff, and Faculty Perspectives on Intimate Partner and Sexual Violence on 3 Public University Campuses: Protocol for the UC Speaks Up Study and Preliminary Results
Source: JMIR Res Protoc. 2022 Apr 5;11(4):e31189. doi: 10.2196/31189 (PMC9019617; doi:10.2196/31189)
Supplement: Multimedia Appendix 1 [file resprot_v11i4e31189_app1.pdf]

Appendix 1

Main Survey

Study ID	__________________________________
Email Address (please provide UC.edu address only)	__________________________________<br>(Your $5 Amazon gift code will be sent here)
How many years old are you?	__________________________________
What is your gender identity?	◯ Male<br>◯ Female<br>◯ Non-binary
What is your racial/ethnic identity?	☐ African American<br>☐ American Indian or Alaska Native<br>☐ Hispanic / LatinX<br>☐ Asian<br>☐ Native Hawaiian or Pacific Islander<br>☐ White<br>(Check all that apply)
Do you consider yourself to be:	◯ Heterosexual or straight<br>◯ Gay or Lesbian<br>◯ Bisexual<br>◯ Other
Level	◯ Undergraduate<br>◯ Postbacc<br>◯ Graduate<br>◯ Professional school (e.g., Medical School, Nursing School, Dental School, Law School)
Field / Discipline / Major	◯ Architecture<br>◯ Arts and Humanities<br>◯ Business<br>◯ Education<br>◯ Engineering/CS<br>◯ Life Sciences<br>◯ Other Health Science<br>◯ Physical Sciences<br>◯ Public Admin<br>◯ Social Sciences<br>◯ Multiple Majors<br>◯ Other/Interdisciplinary<br>◯ Undeclared
Residency Status	◯ California resident<br>◯ Non-resident domestic<br>◯ Non-resident international

Housing	◯ On-campus housing<br>◯ Greek housing (fraternity, sorority, satellite house)<br>◯ Living with a dating/marital/romantic partner<br>◯ Homeless or couch-surfing<br>◯ Living at home with parent(s) or guardian(s)<br>◯ Other living situation
Are you a member of any student groups?	☐ Academic<br>☐ Cultural<br>☐ Art Music or Dance<br>☐ Health professions<br>☐ Sports or athletics<br>☐ Media<br>☐ Service<br>☐ Pre-professional club or Greek<br>☐ Spiritual<br>☐ Campus leadership<br>☐ Social Greek<br>(Check all that apply)

Thank you!

Next we have some questions about how
students in your campus community understand
consent. Remember, we are NOT asking about your personal perspective!

How do students in your campus community know that THEIR PARTNER is signaling consent?	1- the least important way of knowing	2	3	4	5	6	7- the most important way of knowing
verbal consent	○	○	○	○	○	○	○
they said "yes"	○	○	○	○	○	○	○
body language	○	○	○	○	○	○	○
physical cues	○	○	○	○	○	○	○
nonverbal cues	○	○	○	○	○	○	○
reciprocating actions	○	○	○	○	○	○	○
sober	○	○	○	○	○	○	○
not resisting	○	○	○	○	○	○	○
kissing	○	○	○	○	○	○	○
feeling a vibe	○	○	○	○	○	○	○
facial expression	○	○	○	○	○	○	○
not saying no	○	○	○	○	○	○	○
hints	○	○	○	○	○	○	○
touching	○	○	○	○	○	○	○
assumption based on long term relationship	○	○	○	○	○	○	○
nodding	○	○	○	○	○	○	○
going somewhere private	○	○	○	○	○	○	○
having a conversation	○	○	○	○	○	○	○
flirting	○	○	○	○	○	○	○
dancing	○	○	○	○	○	○	○
not pushing you away	○	○	○	○	○	○	○
physically close	○	○	○	○	○	○	○
undressing	○	○	○	○	○	○	○

How do students in your campus community signal THEIR OWN consent?

	1- the least important way of knowing	2	3	4	5	6	7- the most important way of knowing
verbal consent	○	○	○	○	○	○	○
they said "yes"	○	○	○	○	○	○	○
flirting	○	○	○	○	○	○	○
body language	○	○	○	○	○	○	○
having a conversation	○	○	○	○	○	○	○
nonverbal cues	○	○	○	○	○	○	○
physical cues	○	○	○	○	○	○	○
touching	○	○	○	○	○	○	○
initiating	○	○	○	○	○	○	○
reciprocating actions	○	○	○	○	○	○	○
undressing	○	○	○	○	○	○	○
assumption based on long term relationship	○	○	○	○	○	○	○
going somewhere private	○	○	○	○	○	○	○
feeling a vibe	○	○	○	○	○	○	○
sober	○	○	○	○	○	○	○
facial expression	○	○	○	○	○	○	○
hints	○	○	○	○	○	○	○
implied on social media	○	○	○	○	○	○	○
met on a dating app	○	○	○	○	○	○	○
kissing	○	○	○	○	○	○	○
nodding	○	○	○	○	○	○	○
close proximity	○	○	○	○	○	○	○
texting about it ahead of time	○	○	○	○	○	○	○
showing interest	○	○	○	○	○	○	○
reciprocation	○	○	○	○	○	○	○
going on a date	○	○	○	○	○	○	○
laughing at their jokes	○	○	○	○	○	○	○

What words would students in your campus community use to describe a sexual encounter that feels good?

	1- the least important quality	2	3	4	5	6	7- the most important quality
consensual	○	○	○	○	○	○	○
pleasurable	○	○	○	○	○	○	○
they knew what they were doing	○	○	○	○	○	○	○
open communication	○	○	○	○	○	○	○
comfortable	○	○	○	○	○	○	○
they made me cum	○	○	○	○	○	○	○
no guilt	○	○	○	○	○	○	○
meaningful	○	○	○	○	○	○	○
exciting	○	○	○	○	○	○	○
hot	○	○	○	○	○	○	○
considerate	○	○	○	○	○	○	○
passionate	○	○	○	○	○	○	○
safe	○	○	○	○	○	○	○
intimate	○	○	○	○	○	○	○
connection	○	○	○	○	○	○	○
intense	○	○	○	○	○	○	○
respectful	○	○	○	○	○	○	○
kind	○	○	○	○	○	○	○
orgasms	○	○	○	○	○	○	○
sensual	○	○	○	○	○	○	○
in tune	○	○	○	○	○	○	○
